# The Prospective Randomized On-X Valve Anticoagulation Clinical Trial (PROACT): Lower is better, but is it good enough?

**DOI:** 10.21542/gcsp.2019.2

**Published:** 2019-03-31

**Authors:** Ismail Bouhout, Ismail El-Hamamsy

**Affiliations:** Department of Cardiac Surgery, Montreal Heart Institute, Université de Montreal, Quebec, Canada

## Abstract

Due to their durability, mechanical prostheses are frequently used for aortic valve replacement (AVR) in young adults. However, these valves are thrombogenic and require lifelong anticoagulation. Over the last few decades, efforts have been made towards the lowering of INR targets in an effort to reduce bleeding events without influencing the thromboembolic risk. The Prospective Randomized On-X Valve Anticoagulation Clinical Trial (PROACT) was designed to compare standard versus low anticoagulation targets in high-risk patients undergoing mechanical AVR with the ON-X prosthesis.

## Introduction

**Figure 1.**
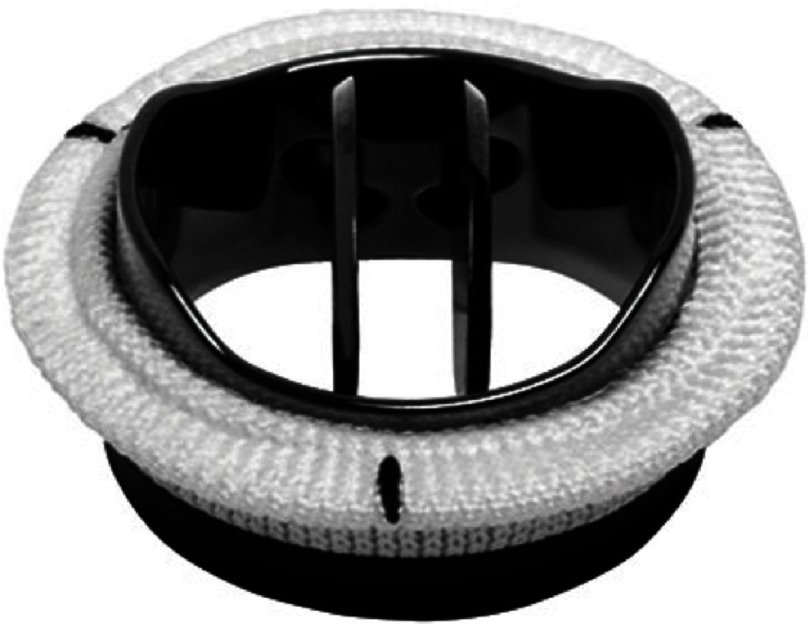
The On-X mechanical prosthesis (Cryolife, USA).

The ideal aortic valve substitute for young adults remains elusive. While bioprostheses avoid long-term anticoagulation, durability is a major concern in young adults because of early structural valve degeneration^1,2^. Due to their durability, mechanical prostheses are more frequently used in young adults. However, these valves are thrombogenic, requiring lifelong anticoagulation which is mostly responsible for valve-related morbidity and mortality following mechanical AVR^2–4^. This partially explains the increased observed mortality in young adults undergoing mechanical AVR in comparison with an age- and gender-matched general population^2^. Therefore, reducing anticoagulation-related complications are major ongoing research objectives to improve survival and quality of life for this patient population. Over the last decades, efforts have been made towards lowering of INR targets, which resulted in a reduction of bleeding-event rates without influencing the risk of thromboembolic (TE) events^5,6^. Additionally, improvements in mechanical prosthesis engineering have led to a substantial reduction in the thrombogenicity of these devices and subsequently a decrease in the incidence of valve-related complications^7^. However, the impact of new bileaflet mechanical prostheses on the safety of lowering anticoagulation targets remains uncertain. The Prospective Randomized On-X Valve Anticoagulation Clinical Trial (PROACT) aimed to evaluate the safety of lower anticoagulation (target INR 1.5–2.0) after implantation of the ON-X prosthesis (Figure 1) in patients at high risk of thromboembolic events^8^.

## The study

The PROACT trial was a multicenter unblended controlled randomized study published in 2014 in *the Journal of Thoracic and Cardiovascular Surgery*^8^. The trial was designed to compare standard versus lower anticoagulation therapy in patients at high risk of TE events, undergoing mechanical AVR with the ON-X prosthesis, including patients with chronic atrial fibrillation, left ventricular ejection fraction <30%, enlarged left atrium (>50 mm), hypercoagulability or resistance to anti-platelet therapy and women receiving estrogen therapy. Ninety days after surgery, 425 patients were enrolled and 375 patients meeting the inclusion criteria were finally randomized in one of two study arms: (1) a targeted INR between 1.5 and 2 (*test group*) and (2) a targeted INR between 2 and 3 *(control group*). Concomitantly, all patients received aspirin 80 mg and were monitored using home INR testing. Any patient who experienced a TE event in the study group was crossed over to the standard INR group, though they remained in the test group through an intention-to-treat analysis. No patients were allowed to cross over from the standard to the test group. The study was sponsored and funded by On-X Life Technologies (Austin, TX) and conducted under an investigational device exemption provided by the US Food and Drug Administration. Primary endpoints were rates of TE events, thrombosis, bleeding and all-cause mortality. An interim non-inferiority analysis was performed for a composite of all primary endpoints, with an absolute margin of 1.5%.

The mean follow-up was 3.8 years and was 98% complete. The mean age was 55 years. The two groups were comparable in terms of patient demographics, except for a marginally higher prevalence of preoperative atrial fibrillation in the control group (6% versus 2%, *p* = 0.06). The mean INR was 1.89 in the test group and 2.5 in the control group (*p* < 0.001).

**Figure 2.**
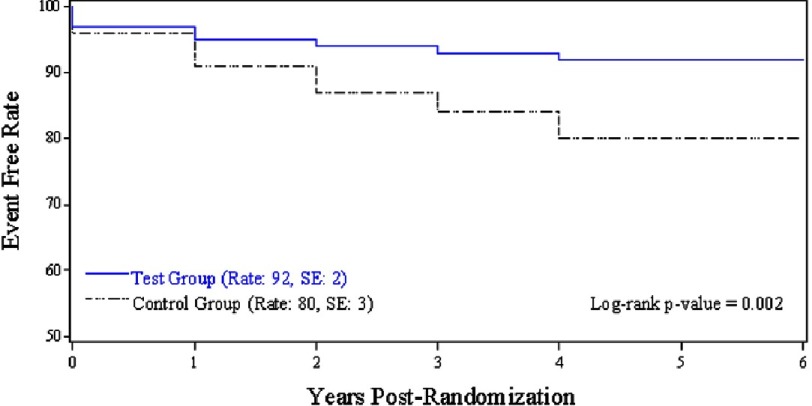
Five-year freedom from major or minor bleeding events in the test group *(blue)* versus the standard group *(black)* in the PROACT trial *(from Puskas et al.*^8^).

**Figure 3.**
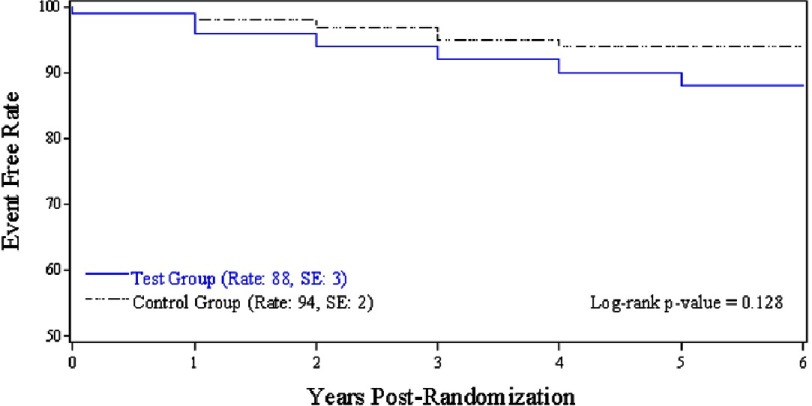
Five-year freedom from thromboembolic events in the test group *(blue)* versus the standard group *(black)* in the PROACT trial *(from Puskas et al.*^8^).

In terms of findings, the composite of major and minor bleeding, TE and thrombosis events was significantly lower in the test group (5.63%/pt-yr in the test group versus 8.47%/pt-yr in the standard group, *p* = 0.046) (Table 1). Furthermore, the test group experienced a significantly lower rate of major bleeding events (1.48%/pt-yr in the test group versus 3.31%/pt-yr in the standard group, *p* = 0.032) and minor bleeding events (1.18%/pt-yr in the test group versus 3.31%/pt-yr in the standard group, *p* = 0.011) (Figure 2), with no differences in the rates of TE and thrombosis events (2.96%/pt-yr in the test group versus 1.85%/pt-yr in the standard group, *p* = 0.178) (Figure 3). Interestingly, when comparing the composite of outcomes while excluding minor events, there was no statistically significant difference between the 2 arms (4.44%/pt-yr in the test group versus 5.16%/pt-yr in the standard group, *p* = 0.539).

**Table 1:** Events rate comparison between patients in the test group and the standard INR group in the PROACT trial (*adapted from Puskas et al.*^8^).

	Test group (%/pt-yr)	Control group (%/pt-yr)	*P* value
Bleeding and TE events	5.63	8.47	0.046
Major events (major bleeding, TE and thrombosis)	4.44	5.16	0.539
Major bleeding events	1.48	3.31	0.032
Minor bleeding events	1.18	3.31	0.011
All bleeding events	2.67	6.62	<0.001
TE and thrombosis events	2.96	1.85	0.178
All cause mortality	1.48	1.46	0.968

**Notes.**

TE thromboembolic

## Discussion

The investigators concluded that, with the On-X prosthesis, a target INR between 1.5 and 2 translates into a lower incidence of bleeding events without a significant increase in TE events. This is consistent with recently published studies demonstrating the safety of a lower anticoagulation regimen in patients with mechanical AVR^9–11^. While the authors state in the limitations that their results could not be extrapolated to other prostheses, the LOWERING-IT trial studied the impact of a lower anticoagulation regimen in patients with various mechanical aortic prostheses^9^. In that study, patients were randomized to low dose INR (1.5–2.5) or standard INR (2–3) anticoagulation therapy. Similar to findings from the PROACT trial, there were no differences in TE events (OR 0.33 [0.006–4.20], *p* = 0.6) while there was a significant decrease in bleeding events in the low-dose group (OR 0.36 [0.11–0.99], *p* = 0.04).

While the PROACT trial was generally well conducted, it raises several important points that warrant consideration. Firstly, the study sample size was calculated to demonstrate non-inferiority of a composite endpoint of bleeding, TE and thrombosis events, which was entirely driven by the reduction in bleeding events in the lower INR group as anticipated. Nevertheless, looking specifically at TE and thrombosis events, there was a 60% higher rate in the test group (2.96%/pt-yr versus 1.85%/pt-yr). Although this did not reach statistical significance (*p* = 0.178), it may be attributed to a lack of statistical power due to sample size.

These findings require further analysis to ensure safety of aiming for lower INR targets.

Secondly, as part of the study protocol, patients were provided a home INR monitoring kit and were closely followed up. This translated into the fact that out of >53,000 measurements, more than 60% of the measured INRs were within the desired range, with 96% of patients having at least one test per month. This correlates with previous studies where home INR monitoring was associated with better INR control, higher long-term survival and lower anticoagulation-related events in patients with mechanical prosthesis^12–14^. However, home INR testing is not widely adopted in the wider population because of availability and cost issues, and patient compliance with testing is overall lower in a real world setting^15^, which results in patients being off range a significant proportion of time. Consequently, results of this trial should be applied with caution in a daily practice because the lower range in the test group (INR 1.5) leaves little safety margin for being under-anticoagulated.

Thirdly, while there was a reduction in anticoagulation-related complications in the low INR group, rates of major bleeding and neurological events (1.48%/patient-year and 1.98%/patient-year, respectively) remain comparable to previously published cohort studies examining long-term outcomes following mechanical AVR^2,16–18^. This suggests that these events may be under-reported and under-estimated in retrospective cohort studies. Importantly, the rates of major events (major bleeding, TE and thrombosis) were not different between the lower and standard anticoagulation groups in the PROACT trial. This has major implications in a young adult population with long anticipated life expectancy where the lifetime risk of experiencing one major anticoagulation-related event is a major consideration.

## What have we learned?

The PROACT trial confirms that a lower anticoagulation target with the On-X prosthesis results in a reduced rate of bleeding and TE. This study represents a major step forward in outcomes improvement for patients with mechanical prostheses. In addition, this study reinforces the potential benefit of home INR monitoring on clinical outcomes in this patient population. Despite a lower INR target and a closer anticoagulation management, anticoagulation-related complications remain the main limitation following mechanical AVR. Furthermore, the real impact of reduced anticoagulation-related events on long-term survival and quality of life of patients, especially in the younger patient population, are yet to be determined. When applying it in clinical practice, this trial should be interpreted in light of recent data available for other valve substitutes. While bioprostheses do not require anticoagulation, durability is a major issue in young adults because of early structural valve degeneration and long-term survival remains sub-optimal^19–21^. Despite these concerns, their use in this population has gained adoption with the advent of transcatheter options for subsequent reintervention^22^.

In contrast, the Ross procedure (pulmonary autograft replacement) alleviates the need for lifelong anticoagulation and is the only operation that guarantees long-term viability of the aortic valve substitute^23^. In several recent reports, this has translated into long-term survival equivalent to the age- and gender-matched general population, a lower risk of valve-related complications and better quality of life than bioprosthetic and mechanical prostheses^24–27^.

**Figure 4.**
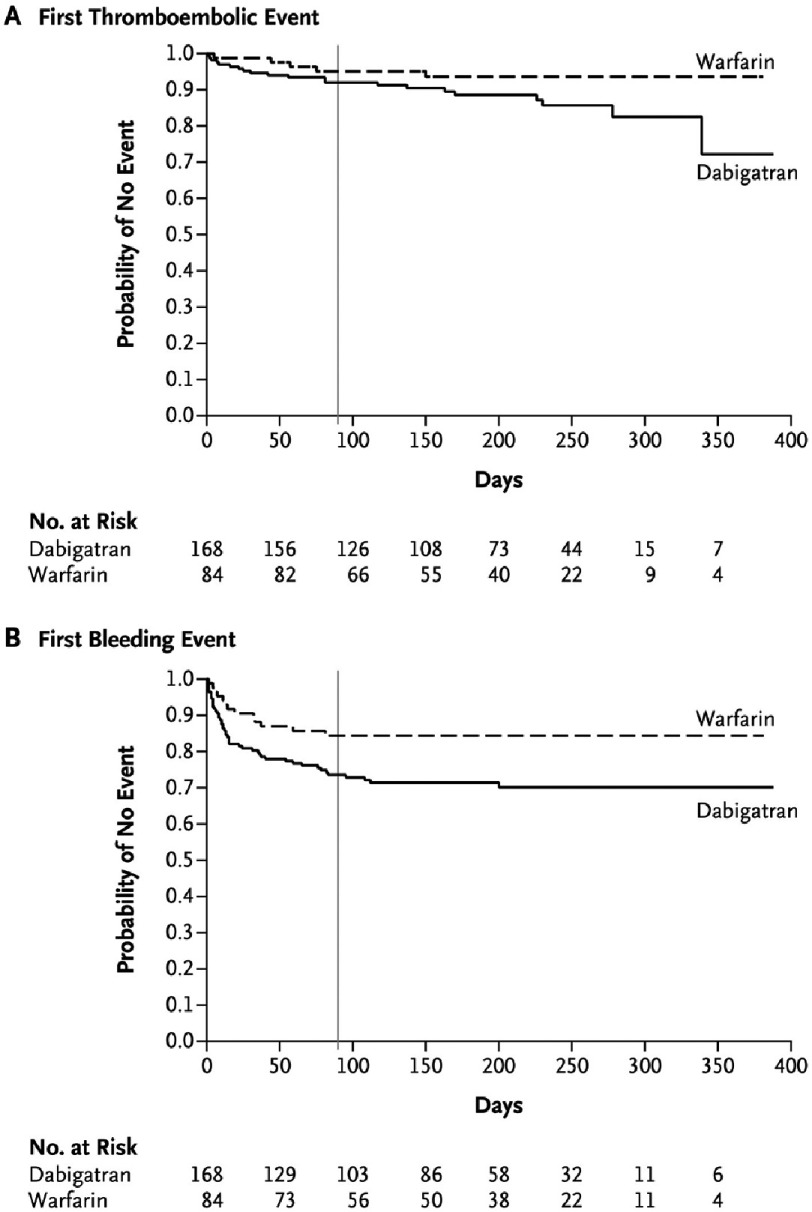
Freedom from (A) thromboembolic and (B) bleeding events following treatment with dabigatran versus warfarin in patients with mechanical heart valves (*from Eikelboom et al.*^28^).

Recently, attempts have been made to broaden anticoagulation options in patients with mechanical prostheses. Novel oral anticoagulants (NOACs) are an alternative to warfarin that preclude the need for laboratory testing. Published in 2013, the RE-ALIGN trial randomized patients to receive Dabigatran versus warfarin after mechanical valve replacement^28^. The use of dabigatran was associated with increased rates of thromboembolic and bleeding events (Figure 4). However, the targeted serum levels of Dabigatran (≥ 50 ng per ml) was extrapolated from studies on stroke prevention in patients with atrial fibrillation and may be insufficient for prosthetic mechanical valves^29^. In addition, all major bleeding events were pericardial effusions occurring in patients who underwent randomization within 1 week following surgery. Therefore, a delayed NOAC initiation strategy may have mitigated the risk of major bleeding. Despite these unfavorable results, ongoing research on NOACs as a lone anticoagulation strategy is currently underway with a phase II study examining the use of Rivaroxaban in patients with mechanical prostheses (CATHAR, NCT02128841). Additionally, a non-inferiority trial comparing a low INR strategy (1.5–2) and NOACs in patients with previous AVR using the On-X prosthesis should be considered.

In conclusion, the PROACT trial demonstrates the safety and feasibility of targeting lower INR values in patients with home anticoagulation monitoring undergoing mechanical AVR using the On-X prosthesis. This represents a significant step forward in patient management, with demonstrable improvements in patient outcomes. Findings from this trial highlight the need for a prospective randomized trial comparing mechanical and bioprosthetic valves to the Ross procedure in young adults undergoing AVR.

## Competing interests

No extra-mural funding supported this work.

**Funding sources**

The authors have no conflicts of interest to disclose.

## References

[1] Forcillo J, El Hamamsy I, Stevens LM, Badrudin D, Pellerin M, Perrault LP, Cartier R, Bouchard D, Carrier M, Demers P (2014). The perimount valve in the aortic position: twenty-year experience with patients under 60 years old. Ann Thorac Surg.

[2] Bouhout I, Stevens LM, Mazine A, Poirier N, Cartier R, Demers P, El-Hamamsy I (2014). Long-term outcomes after elective isolated mechanical aortic valve replacement in young adults. J Thorac Cardiovasc Surg.

[3] Stein PD, Alpert JS, Bussey HI, Dalen JE, Turpie AG (2001). Antithrombotic therapy in patients with mechanical and biological prosthetic heart valves. Chest.

[4] Hammermeister K, Sethi GK, Henderson WG, Grover FL, Oprian C, Rahimtoola SH (2000). Outcomes 15 years after valve replacement with a mechanical versus a bioprosthetic valve: final report of the Veterans Affairs randomized trial. J Am Coll Cardiol.

[5] Altman R, Rouvier J, Gurfinkel E, Scazziota A, Turpie AG (1996). Comparison of high-dose with low-dose aspirin in patients with mechanical heart valve replacement treated with oral anticoagulant. Circulation.

[6] Meschengieser SS, Fondevila CG, Frontroth J, Santarelli MT, Lazzari MA (1997). Low-intensity oral anticoagulation plus low-dose aspirin versus high-intensity oral anticoagulation alone: a randomized trial in patients with mechanical prosthetic heart valves. J Thorac Cardiovasc Surg.

[7] DeWall RA, Qasim N, Carr L (2000). Evolution of mechanical heart valves. Ann Thorac Surg.

[8] Puskas J, Gerdisch M, Nichols D, Quinn R, Anderson C, Rhenman B, Fermin L, McGrath M, Kong B, Hughes C, Sethi G, Wait M, Martin T, Graeve A, Investigators P (2014). Reduced anticoagulation after mechanical aortic valve replacement: interim results from the prospective randomized on-X valve anticoagulation clinical trial randomized Food and Drug Administration investigational device exemption trial. J Thorac Cardiovasc Surg.

[9] Torella M, Torella D, Chiodini P, Franciulli M, Romano G, De Santo L, De Feo M, Amarelli C, Sasso FC, Salvatore T, Ellison GM, Indolfi C, Cotrufo M, Nappi G (2010). LOWERing the INtensity of oral anticoaGulant Therapy in patients with bileaflet mechanical aortic valve replacement: results from the “LOWERING-IT” Trial. Am Heart J.

[10] Koertke H, Zittermann A, Wagner O, Ennker J, Saggau W, Sack FU, Cremer J, Huth C, Braccio M, Musumeci F, Koerfer R (2010). Efficacy and safety of very low-dose self-management of oral anticoagulation in patients with mechanical heart valve replacement. Ann Thorac Surg.

[11] Horstkotte D, Schulte HD, Bircks W, Strauer BE (1994). Lower intensity anticoagulation therapy results in lower complication rates with the St. Jude Medical prosthesis. J Thorac Cardiovasc Surg..

[12] Koertke H, Zittermann A, Wagner O, Koerfer R (2007). Self-management of oral anticoagulation therapy improves long-term survival in patients with mechanical heart valve replacement. Ann Thorac Surg.

[13] Butchart EG, Payne N, Li HH, Buchan K, Mandana K, Grunkemeier GL (2002). Better anticoagulation control improves survival after valve replacement. J Thorac Cardiovasc Surg.

[14] Heneghan C, Ward A, Perera R, Self-Monitoring Trialist C, Bankhead C, Fuller A, Stevens R, Bradford K, Tyndel S, Alonso-Coello P, Ansell J, Beyth R, Bernardo A, Christensen TD, Cromheecke ME, Edson RG, Fitzmaurice D, Gadisseur AP, Garcia-Alamino JM, Gardiner C, Hasenkam JM, Jacobson A, Kaatz S, Kamali F, Khan TI, Knight E, Kortke H, Levi M, Matchar D, Menendez-Jandula B, Rakovac I, Schaefer C, Siebenhofer A, Souto JC, Sunderji R, Gin K, Shalansky K, Voller H, Wagner O, Zittermann A (2012). Self-monitoring of oral anticoagulation: systematic review and meta-analysis of individual patient data. Lancet.

[15] Amara W, Larsen TB, Sciaraffia E, Hernandez Madrid A, Chen J, Estner H, Todd D, Bongiorni MG, Potpara TS, Dagres N, Sagnol P, Blomstrom-Lundqvist C (2016). Patients’ attitude and knowledge about oral anticoagulation therapy: results of a self-assessment survey in patients with atrial fibrillation conducted by the European Heart Rhythm Association. Europace.

[16] Ikonomidis JS, Kratz JM, Crumbley 3rd AJ, Stroud MR, Bradley SM, Sade RM, Crawford Jr FA (2003). Twenty-year experience with the St Jude Medical mechanical valve prosthesis. J Thorac Cardiovasc Surg.

[17] Khan SS, Trento A, DeRobertis M, Kass RM, Sandhu M, Czer LS, Blanche C, Raissi S, Fontana GP, Cheng W, Chaux A, Matloff JM (2001). Twenty-year comparison of tissue and mechanical valve replacement. J Thorac Cardiovasc Surg.

[18] Ruel M, Kulik A, Lam BK, Rubens FD, Hendry PJ, Masters RG, Bedard P, Mesana TG (2005). Long-term outcomes of valve replacement with modern prostheses in young adults. European Journal of Cardio-thoracic Surgery: Official Journal of the European Association for Cardio-thoracic Surgery.

[19] Welke KF, Wu Y, Grunkemeier GL, Ahmad A, Starr A (2011). Long-term results after Carpentier-Edwards pericardial aortic valve implantation, with attention to the impact of age. The heart surgery forum.

[20] McClure RS, Narayanasamy N, Wiegerinck E, Lipsitz S, Maloney A, Byrne JG, Aranki SF, Couper GS, Cohn LH (2010). Late outcomes for aortic valve replacement with the Carpentier-Edwards pericardial bioprosthesis: up to 17-year follow-up in 1,000 patients. Ann Thorac Surg.

[21] Forcillo J, Pellerin M, Perrault LP, Cartier R, Bouchard D, Demers P, Carrier M (2013). Carpentier-Edwards pericardial valve in the aortic position: 25-years experience. Ann Thorac Surg.

[22] Dvir D, Webb J, Brecker S, Bleiziffer S, Hildick-Smith D, Colombo A, Descoutures F, Hengstenberg C, Moat NE, Bekeredjian R, Napodano M, Testa L, Lefevre T, Guetta V, Nissen H, Hernandez JM, Roy D, Teles RC, Segev A, Dumonteil N, Fiorina C, Gotzmann M, Tchetche D, Abdel-Wahab M, De Marco F, Baumbach A, Laborde JC, Kornowski R (2012). Transcatheter aortic valve replacement for degenerative bioprosthetic surgical valves: results from the global valve-in-valve registry. Circulation.

[23] Rabkin-Aikawa E, Aikawa M, Farber M, Kratz JR, Garcia-Cardena G, Kouchoukos NT, Mitchell MB, Jonas RA, Schoen FJ (2004). Clinical pulmonary autograft valves: pathologic evidence of adaptive remodeling in the aortic site. J Thorac Cardiovasc Surg.

[24] El-Hamamsy I, Eryigit Z, Stevens LM, Sarang Z, George R, Clark L, Melina G, Takkenberg JJ, Yacoub MH (2010). Long-term outcomes after autograft versus homograft aortic root replacement in adults with aortic valve disease: a randomised controlled trial. Lancet.

[25] David TE, David C, Woo A, Manlhiot C (2014). The Ross procedure: outcomes at 20 years. J Thorac Cardiovasc Surg.

[26] Sievers HH, Stierle U, Charitos EI, Hanke T, Gorski A, Misfeld M, Bechtel M (2010). Fourteen years’ experience with 501 subcoronary Ross procedures: surgical details and results. J Thorac Cardiovasc Surg.

[27] El-Hamamsy I, Poirier NC (2013). What is the role of the Ross procedure in today’s armamentarium?. Can J Cardiol.

[28] Eikelboom JW, Connolly SJ, Brueckmann M, Granger CB, Kappetein AP, Mack MJ, Blatchford J, Devenny K, Friedman J, Guiver K, Harper R, Khder Y, Lobmeyer MT, Maas H, Voigt JU, Simoons ML, Van de Werf F, Investigators R-A (2013). Dabigatran versus warfarin in patients with mechanical heart valves. N Engl J Med.

[29] Jaffer IH, Stafford AR, Fredenburgh JC, Whitlock RP, Chan NC, Weitz JI (2015). Dabigatran is less effective than warfarin at attenuating mechanical heart valve-induced thrombin generation. J Am Heart Assoc.

